# Comparison of Clinical Outcomes Following Lumbar Endoscopic Unilateral Laminotomy Bilateral Decompression and Minimally Invasive Transforaminal Lumbar Interbody Fusion for One-Level Lumbar Spinal Stenosis With Degenerative Spondylolisthesis

**DOI:** 10.3389/fsurg.2020.596327

**Published:** 2021-02-26

**Authors:** Wenbin Hua, Bingjin Wang, Wencan Ke, Qian Xiang, Xinghuo Wu, Yukun Zhang, Shuai Li, Shuhua Yang, Qiang Wu, Cao Yang

**Affiliations:** Department of Orthopaedics, Union Hospital, Tongji Medical College, Huazhong University of Science and Technology, Wuhan, China

**Keywords:** lumbar endoscopic unilateral laminotomy bilateral decompression, minimally invasive, transforaminal lumbar interbody fusion, lumbar spinal stenosis, degenerative spondylolisthesis

## Abstract

**Introduction:** Both lumbar endoscopic unilateral laminotomy bilateral decompression (LE-ULBD) and minimally invasive transforaminal lumbar interbody fusion (MI-TLIF) have been used to treat one-level lumbar spinal stenosis (LSS) with degenerative spondylolisthesis, while the differences of the clinical outcomes are still uncertain.

**Methods:** Among 60 consecutive patients included, 24 surgeries were performed by LE-ULBD and 36 surgeries were performed by MI-TLIF. Patient demographics, operation characteristics and complications were recorded. Sagittal parameters, including slip percentage (SP) and slip angle (SA) were compared. The visual analog scale (VAS) score, the Oswestry Disability Index (ODI) score, and Macnab criteria were used to evaluate the clinical outcomes. Follow-up examinations were conducted at 3, 6, 12, and 24 months postoperatively.

**Results:** The estimated blood loss, time to ambulation and length of hospitalization of the LE-ULBD group were shorter than the MI-TLIF group. Preoperative and final follow-up SP of the LE-ULBD group was of no significant difference, while final follow-up SP of the MI-TLIF group was significantly improved compared with preoperative SP. The postoperative mean VAS and ODI scores decreased significantly in both LE-ULBD group and MI-TLIF group. According to the modified Macnab criteria, the outcomes rated as excellent/good rate were 95.8 and 97.2%, respectively, in both LE-ULBD group and MI-TLIF group. Intraoperative complication rate of the LE-ULBD and the MI-TLIF group were 4.2 and 0%, respectively. One case of intraoperative epineurium injury was observed in the LE-ULBD group. Postoperative complication rate of the LE-ULBD and the MI-TLIF group were 0 and 5.6%, respectively. One case with transient urinary retention and one case with pleural effusion were observed in the MI-TLIF group.

**Conclusion:** Both LE-ULBD and MI-TLIF are safe and effective to treat one-level LSS with degenerative spondylolisthesis.

## Introduction

Lumbar spinal stenosis (LSS) with degenerative spondylolisthesis is an important cause of low back pain (1). The optimal treatment for patients of LSS with degenerative spondylolisthesis remains uncertain (2, 3). Surgery may be necessary to relieve the symptoms and improve function after failed conservative treatment in patients of LSS with degenerative spondylolisthesis (4, 5). Decompression alone, such as laminotomy with medial facetectomy, may result in segmental spinal instability (6). Decompression with instrumented fusion was thought as the “criterion standard” treatment for LSS with degenerative spondylolisthesis (7–9). Minimally invasive transforaminal lumbar interbody fusion (MI-TLIF) was firstly described by Foley et al. and had been commonly performed to treat LSS with degenerative spondylolisthesis (10–12). Decompression alone was also used to treat LSS with degenerative spondylolisthesis to preserve the facet joints, paraspinal musculoligamentous structures, mitigate the risk of adjacent segment fusions after instrumented fusion, and to reduce the healthcare cost (2). Minimally invasive unilateral laminotomy bilateral decompression was performed to treat LSS with degenerative spondylolisthesis to minimize the injury to the paraspinal musculoligamentous structures (6). Lumbar endoscopic unilateral laminotomy bilateral decompression (LE-ULBD) has been used to treat LSS with degenerative spondylolisthesis to further minimize the injury to the paraspinal musculoligamentous structures in recent years (6, 13–17).

Even though both decompression alone and decompression plus instrumented fusion has been performed to treat patients of LSS with degenerative spondylolisthesis, it is controversial about the necessity of instrumented fusion after decompression (2, 7, 8, 12). The purpose of the present retrospective study is to compare the clinical outcomes, safety and complications of LE-ULBD and MI-TLIF to treat one-level LSS with degenerative spondylolisthesis.

## Materials and Methods

### Ethics Statement

The present study was conducted in accordance with the guidelines of the Declaration of Helsinki and was approved by the ethics committee of Tongji Medical College, Huazhong University of Science and Technology. Written informed consent was obtained from the individuals for the publication of any potentially identifiable images or data included in this article.

### Patient Population and Grouping

This retrospective study included 60 patients (18 males, 42 females) of LSS with degenerative spondylolisthesis, who underwent LE-ULBD or MI-TLIF in our department between January 2016 and December 2017. An informed consent has been obtained from each patient included. In these patients, 24 surgeries were performed by LE-ULBD and the other 36 surgeries were performed by MI-TLIF.

Inclusion criteria were as follows: patients with typical symptoms, such as leg pain, numbness, motor weakness, neurogenic claudication or radiculopathy; lateral, flexion, and extension radiographs indicating grade 1 degenerative spondylolisthesis; computed tomography (CT) and magnetic resonance imaging (MRI) indicating one-level LSS with grade 1 degenerative spondylolisthesis, in agreement with clinical symptoms and signs; a history of failed conservative treatment for more than 3 months or progressive neurological symptoms; and follow-up for at least 24 months. Exclusion criteria were as follows: patients with radiographic confirmation of isthmic spondylolisthesis; lumbar pathologies requiring surgery at two or more levels; previous lumbar fusion surgery; and tumors, infections, or other lesions (6, 10, 11, 17). Each patient that met all the criteria underwent LE-ULBD or MI-TLIF under general anesthesia.

Decision-making criteria: One-level LSS with grade 1 degenerative spondylolisthesis could be treated by either LE-ULBD and MI-TLIF. Patients without facet joint diastasis, significant motion on flexion and extension radiographs, were thought as the surgical indication for LE-ULBD (2). However, patients with facet joint diastasis, or significant motion on flexion and extension radiographs, indicating potential dynamic instability were recommend to undergo MI-TLIF (2). Radiographic dynamic instability was defined as lumbar segmental translational motion >3 mm on lateral flexion-extension flexion and extension radiographs (17, 18).

### Surgical Technique

All the surgeries were performed by the senior author. Both LE-ULBD and MI-TLIF were performed under general anesthesia with the patient in the prone position.

#### LE-ULBD

**Figure 1:**
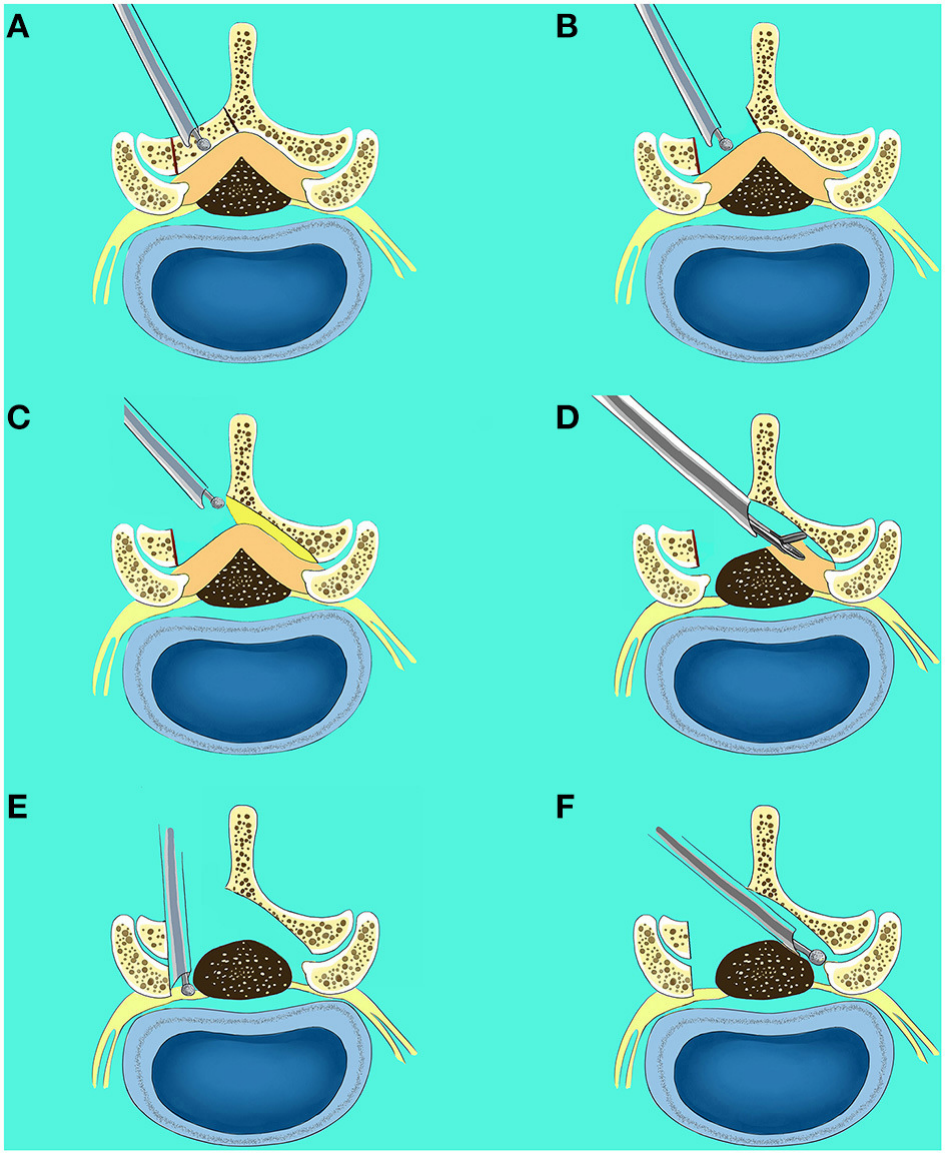
Surgical procedures of lumbar endoscopic unilateral laminotomy bilateral decompression (LE-ULBD). **(A,B)** The inferior edge of the cranial lamina and the base of the spinous process of the ipsilateral side were removed by the endoscopic burr; **(C)** undercutting of the contralateral cranial lamina was performed; **(D)** the ipsilateral and contralateral ligamentum flavum was identified and removed piecemeal with endoscopic punches and forceps; **(E)** the ipsilateral medial facetectomy was performed to decompress the lateral recess and ensure adequate decompression of the traversing nerve root; **(F)** the contralateral medial facetectomy was performed to decompress the lateral recess and ensure adequate decompression of the traversing nerve root.

**Figure 2:**
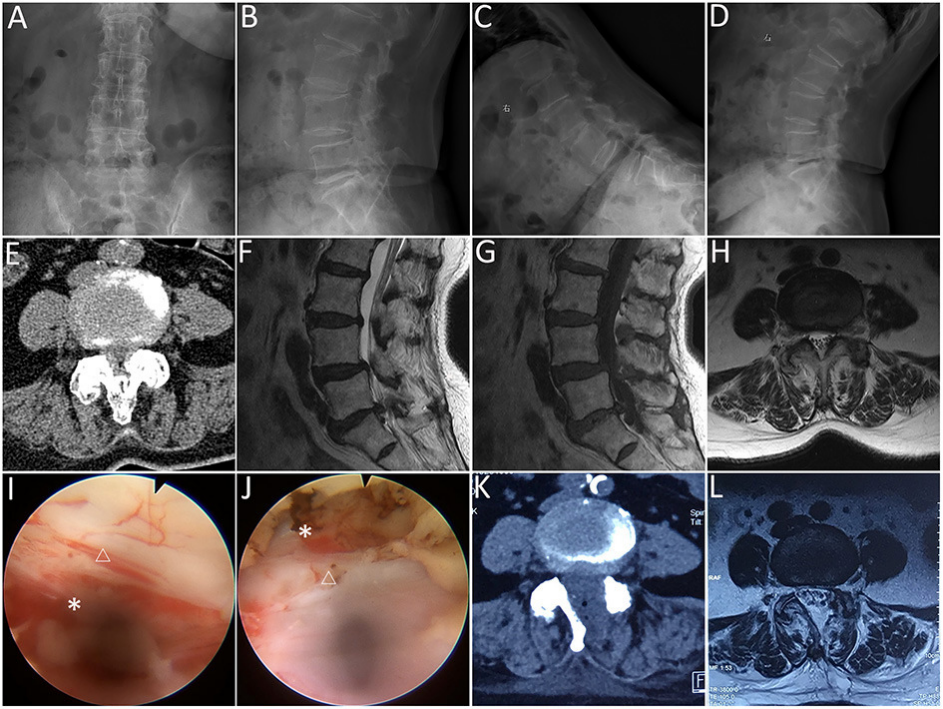
Lumbar endoscopic unilateral laminotomy bilateral decompression (LE-ULBD) performed on a 77-year-old female patient diagnosed with L4-L5 lumbar spinal stenosis with degenerative spondylolisthesis. **(A,B)** preoperative anteroposterior and lateral plain radiographs; **(C,D)** preoperative flexion and extension radiographs; **(E)** preoperative computed tomography (CT) scans; **(F–H)** preoperative magnetic resonance imaging (MRI) scans; **(I,J)** medial facetectomy was performed to decompress the lateral recess and ensure adequate decompression of the traversing nerve root; **(K)** postoperative CT scans; **(L)** postoperative MRI scans. Snowflake, nerve root, triangle, dural sac. * is used to tell the readers where is the nerve root.

Posteroanterior and lateral fluoroscopy were used to locate the interlaminar space at the surgery segment. A 10 mm skin incision was lateral to the outer border of the interlaminar window. Soft tissue expanders were applied *via* the incision to assist the insertion of the working sheath and the endoscopic surgical system (Spinendos, Munich, Germany). All the subsequent procedures were performed under constant irrigation with excellent endoscopic visualization. The inferior edge of the cranial lamina and the base of the spinous process of the ipsilateral side were removed by the endoscopic burr (Spinendos), enabling access into the spinal canal. Once the epidural space was entered, undercutting of the contralateral cranial lamina was performed. Then the ipsilateral and contralateral ligamentum flavum was identified and removed piecemeal with endoscopic punches and forceps. To minimize dural and epineural injury, the contralateral ligamentum flavum can be removed at first, then the ipsilateral ligamentum flavum is removed. Subsequently, ipsilateral and contralateral medial facetectomy was performed to decompress the lateral recess and ensure adequate decompression of the traversing nerve root. Once the traversing nerve root was decompressed, it was reflected medially using a blunt dissector. The bipolar radiofrequency electrocoagulator (Trigger-Flex; Elliquence, Baldwin, NY, USA) was used for hemostasis, soft-tissue clearance and adhesion release. Prior to surgery completion, we ensured there was no significant dural sac damage or active bleeding. No drainages were required. These procedures were presented in Figure 1. A representative case is shown in Figure 2.

#### MI-TLIF

**Figure 3:**
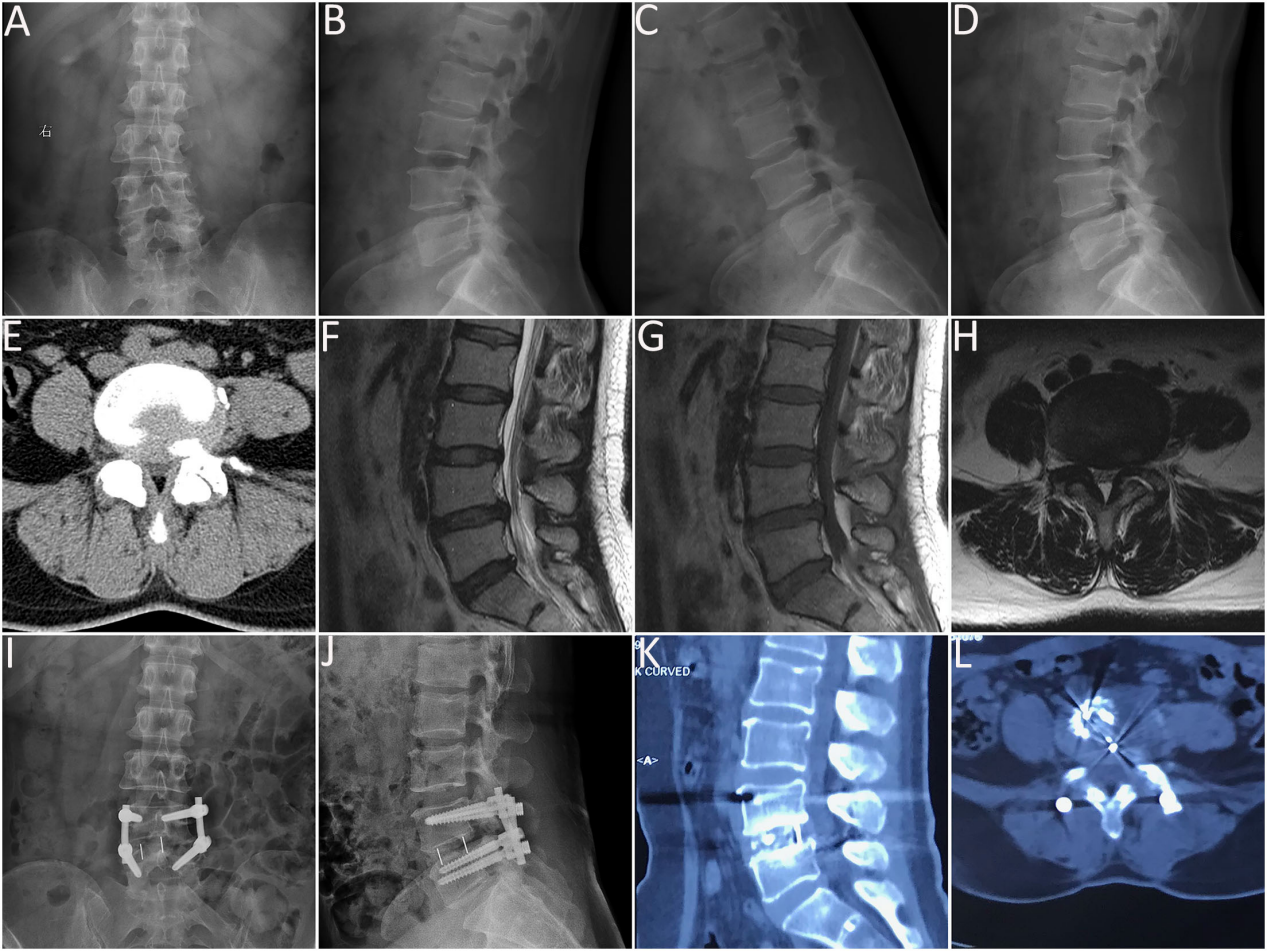
Minimally invasive transforaminal lumbar interbody fusion (MI-TLIF) performed on a 48-year-old female patient diagnosed with L4-L5 lumbar spinal stenosis with degenerative spondylolisthesis. **(A,B)** preoperative anteroposterior and lateral plain radiographs; **(C,D)** preoperative flexion and extension radiographs; **(E)** preoperative computed tomography (CT) scans; **(F–H)** preoperative magnetic resonance imaging (MRI) scans; **(I,J)** anteroposterior and lateral plain radiographs 3 days after the surgery; **(K,L)** CT scans 12 months after the surgery.

Posteroanterior and lateral fluoroscopy were used to locate the pedicles of the surgery level. The Wiltse approach was undertaken through a paramedian skin incision. A Quadrant tubular dilator (Medtronic Sofamor Danek USA Inc, MN, USA) was used for unilateral facet exposure. Facetectomy was performed on the ipsilateral side in order to visualize the transforaminal disc space. Laminectomy and lateral recess decompression were performed to decompress the spinal canal. Besides, the tubular retractor could be angled medially to complete a more extensive decompression of central canal stenosis and the contralateral side. Ligamentum flavum was adequately resected to expose the ipsilateral traversing and exiting nerve roots. A standard discectomy and endplates removal were performed. The autogenous and allogeneic (Aorui, China) bone graft was placed anteriorly and contralateral to the annulotomy, then an intervertebral cage filled with autogenous and allogeneic bone graft was placed. In addition, unilateral pedicle screws were placed ipsilateral to the approach, and contralateral pedicle screws were placed through a contralateral incision. Rods were sized appropriately and tunneled through the paramedian incisions. The incisions were irrigated and closed in layers with drainages kept for no more than 48 h. A representative case is shown in Figure 3.

### Clinical Evaluation

Patient demographic, past medical history, symptom and signs were collected and summarized. The operation time, estimated blood loss, time to ambulation, length of hospitalization, intraoperative and postoperative complications were recorded. The healthcare cost was also recorded.

**Figure 4:**
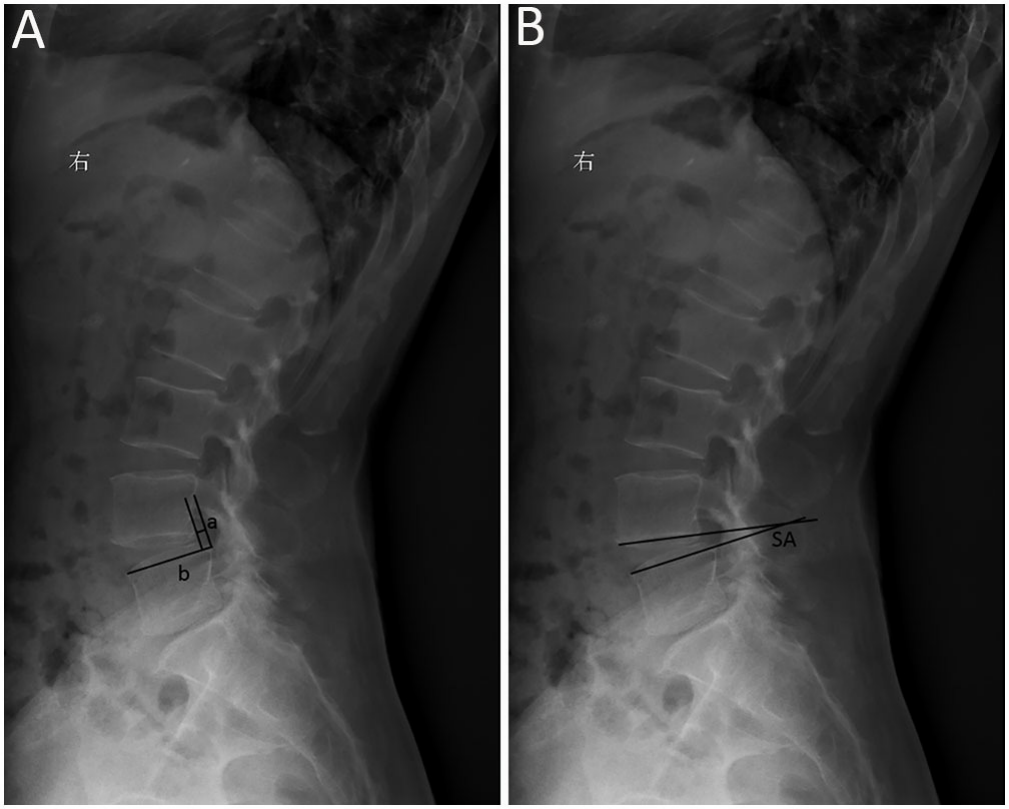
Sagittal parameters of degenerative spondylolisthesis. **(A)** Slip percentage (SP) was measured as a percentage of the distance from the posterior border of the caudal vertebra to the posterior border of the cephalic vertebra (a), normalized to the superior endplate diameter of the caudal vertebra (b); SP = a/b × 100%; **(B)** slip angle (SA) was measured by Cobb's angle between the inferior endplate of the cephalic vertebra and superior endplate of caudal vertebra.

Follow-up examinations were conducted at 3, 6, 12, and 24 months postoperatively. Plain radiography was performed preoperatively and postoperatively at follow-up time points. Preoperative and final follow-up sagittal parameters, including slip percentage (SP) and slip angle (SA) were compared (Figure 4). SP was defined as a percentage of the distance from the posterior border of the caudal vertebra to the posterior border of the cephalic vertebra, normalized to the superior endplate diameter of the caudal vertebra; and SA was defined by Cobb's angle between the inferior endplate of the cephalic vertebra and superior endplate of caudal vertebra (19). MRI or CT was performed preoperatively and postoperatively in necessary. Patient-reported outcomes, including visual analog scale (VAS) score for leg pain and back pain (0–10), Oswestry Disability Index (ODI) score (range, 0–100) and modified Macnab criteria, were recorded preoperatively and postoperatively at follow-up time points.

### Statistical Analyses

All data are presented as mean ± standard deviation. SPSS 22.0 (IBM Corp., Armonk, NY, USA) was used to perform the statistical analyses. GraphPad Prism 6 (Graph Pad Software, Inc., San Diego, CA, USA) was used to generate plots. Non-parametric data was analyzed by Mann-Whitney U test or Wilcoxon signed-rank test. A *p* < 0.05 was considered statistically significant.

## Results

### Demographic Data

Twenty-four patients were included in the LE-ULBD group, and the other 36 patients were included in the MI-TLIF group. The clinical characteristics of both groups are summarized in Table 1. There were no significant differences in sex, age, levels involved and preoperative symptoms among both groups.

**Table 1:** Baseline characteristics of the two groups.

	**LE-ULBD**	**MI-TLIF**	***P*-value**
*N*	24	36	–
Male/female	8/16	10/26	0.648
Age (years)	59.0 ± 7.9 (41–77)	59.9 ± 8.6 (41–81)	0.540
**Levels involved**			
L3-L4	1 (4.2%)	1 (2.8%)	0.520
L4-L5	18 (75.0%)	25 (69.4%)	
L5-S1	5 (20.8%)	10 (27.8%)	
**Preoperative symptoms**			
Back pain	21 (87.5%)	32 (88.9%)	0.871
Leg pain	22 (91.7%)	34 (94.4%)	0.675
Numbness	19 (79.2%)	27 (75.0%)	0.711
Motor weakness	17 (70.8%)	25 (69.4%)	0.909

*N indicates number of patients included in the statistical analysis; LE-ULBD, lumbar endoscopic unilateral laminotomy bilateral decompression; MI-TLIF, minimally invasive transforaminal lumbar interbody fusion*.

### Clinical Outcomes

The mean operation time was 142.5 ± 34.2 min in the LE-ULBD group, 158.0 ± 42.8 min in the MI-TLIF group (*P* = 0.183). The estimated blood loss, time to ambulation and length of hospitalization of the LE-ULBD group were shorter than the MI-TLIF group (Table 2).

**Table 2:** Operation characteristics of the two groups.

	**LE-ULBD**	**MI-TLIF**	***P*-value**
*N*	24	36	–
Operation time (min)	142.5 ± 34.2 (85–240)	158.0 ± 42.8 (60–270)	0.183
Estimated blood loss (ml)	50.4 ± 10.8 (40–80)	149.4 ± 89.8 (50–400)	<0.001
Time to ambulation (h)	12.0 ± 4.0 (8–28)	22.7 ± 10.2 (12–48)	<0.001
Length of hospitalization (d)	2.6 ± 1.0 (1–4)	11.1 ± 2.6 (7–17)	<0.001

*N indicates number of patients included in the statistical analysis; LE-ULBD, lumbar endoscopic unilateral laminotomy bilateral decompression; MI-TLIF, minimally invasive transforaminal lumbar interbody fusion*.

Preoperative SP and SA of both groups were of no significant difference. Final follow-up SP was 9 ± 4 in the LE-ULBD group, 3 ± 3 in the MI-TLIF group (*P* < 0.001). Final follow-up SP of the LE-ULBD group was of no significant difference with preoperative SP, while final follow-up SP of the MI-TLIF group was significantly improved compared with preoperative SP. Final follow-up SA was 8 ± 3 in the LE-ULBD group, 8 ± 4 in the MI-TLIF group (*P* = 0.827). Final follow-up SA of both groups were of no significant difference with preoperative SA (Table 3).

**Table 3:** Radiologic evaluation of the two groups.

	**LE-ULBD**	**MI-TLIF**	***P*-value**
*N*	24	36	–
Preoperative SP(%)	8 ± 3 (3–12)	10 ± 3 (5–16)	0.077
Final follow-up SP(%)	9 ± 4 (3–14)^#^	3 ± 3 (0–7)^*^	<0.001
Preoperative SA(°)	8 ± 2 (6–13)	6 ± 3 (2–10)	0.126
Final follow-up SA(°)	8 ± 3 (6–14)^#^	8 ± 4 (2–12)^#^	0.827

*N indicates number of patients included in the statistical analysis; LE-ULBD, lumbar endoscopic unilateral laminotomy bilateral decompression; MI-TLIF, minimally invasive transforaminal lumbar interbody fusion; SP, slip percentage; SA, slip angle;*

* *P < 0.05;*

# *P > 0.05*.

**Figure 5:**
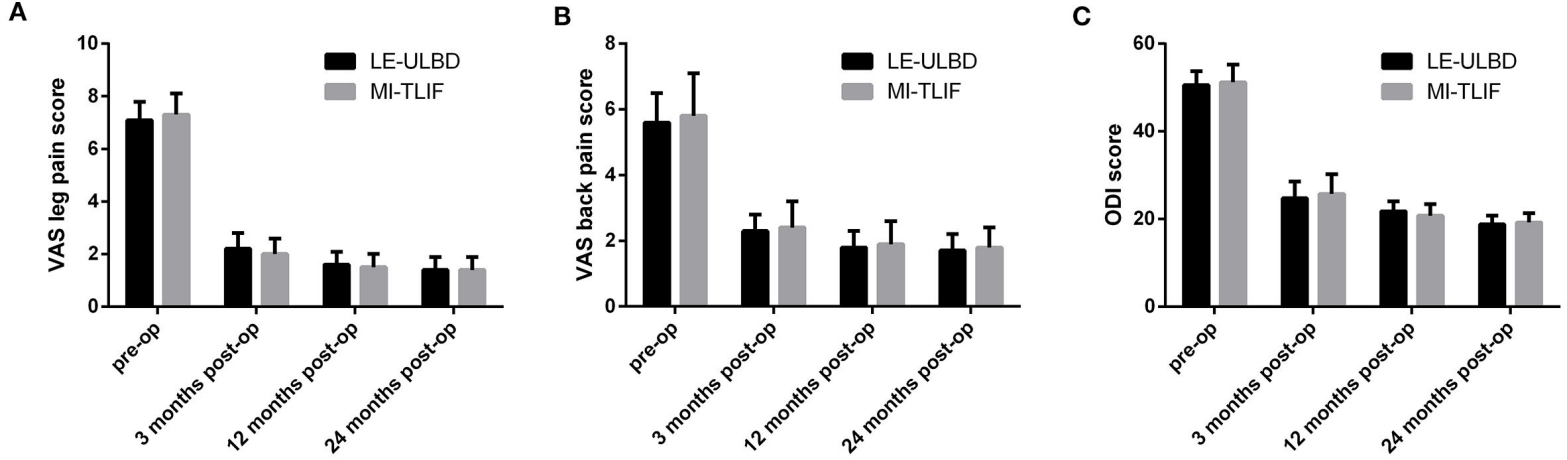
The mean visual analog scale (VAS) scores for leg and back pain, and Oswestry disability index (ODI) scores. **(A)** VAS scores for leg pain; **(B)** VAS scores for back pain; **(C)** ODI scores. Pre-op, pre-operative; post-op, post-operative; LE-ULBD, lumbar endoscopic unilateral laminotomy bilateral decompression; MI-TLIF, minimally invasive transforaminal lumbar interbody fusion.

Preoperative mean VAS scores and ODI scores of both groups were of no significant difference. The mean VAS scores and ODI scores improved significantly postoperatively in both LE-ULBD and MI-TLIF groups (Table 4). The mean VAS scores and ODI scores of the both groups were of no significant difference (Figure 5).

**Table 4:** Comparison of VAS and ODI scores in the two groups.

		**LE-ULBD**	**MI-TLIF**	***P*-value**
*N*		24	36	–
VAS leg pain	Pre-op	7.1 ± 0.7	7.3 ± 0.8	0.455
	3 months post-op	2.2 ± 0.6^*^	2.0 ± 0.6^*^	0.502
	12 months post-op	1.6 ± 0.5^*^	1.5 ± 0.5^*^	0.460
	24 months post-op	1.4 ± 0.5^*^	1.4 ± 0.5^*^	0.667
VAS back pain	Pre-op	5.6 ± 0.9	5.8 ± 1.3	0.567
	3 months post-op	2.3 ± 0.5^*^	2.4 ± 0.8^*^	0.722
	12 months post-op	1.8 ± 0.5^*^	1.9 ± 0.7^*^	0.537
	24 months post-op	1.7 ± 0.5^*^	1.8 ± 0.6^*^	0.576
ODI (%)	Pre-op	50.6 ± 3.2	51.2 ± 3.4	0.457
	3 months post-op	24.8 ± 3.8^*^	25.7 ± 4.5^*^	0.539
	12 months post-op	21.8 ± 2.2^*^	20.8 ± 2.6^*^	0.142
	24 months post-op	18.8 ± 2.0^*^	19.2 ± 2.1^*^	0.383

*N indicates number of patients included in the statistical analysis; LE-ULBD, lumbar endoscopic unilateral laminotomy bilateral decompression; MI-TLIF, minimally invasive transforaminal lumbar interbody fusion; pre-op, preoperative; post-op, postoperative; VAS, Visual Analog Scale; ODI, Oswestry Disability Index*.

* *P < 0.05 vs. preoperative data*.

According to the modified Macnab criteria, the outcomes rated as excellent/good rate were 95.8 and 97.2% in the two groups (Table 5).

**Table 5:** Comparison of modified Macnab evaluation in the two groups.

**Modified Macnab evaluation**		**LE-ULBD**	**MI-TLIF**
Outcome	Excellence (N)	14	21
	Good (N)	9	14
	Fair (N)	1	1
	Poor (N)	0	0
Excellence/good rate (%)		95.8	97.2

*N indicates number of patients included in the statistical analysis; LE-ULBD, lumbar endoscopic unilateral laminotomy bilateral decompression; MI-TLIF, minimally invasive transforaminal lumbar interbody fusion*.

### Complications

Intraoperative and postoperative complications of LE-ULBD and MI-TLIF group were also compared (Table 6). Intraoperative complication rate of the LE-ULBD and the MI-TLIF group were 4.2 and 0%, respectively (*P* = 0.221). One case of intraoperative epineurium injury was observed in the LE-ULBD group. No nerve injury, dural injury, or cauda equina syndrome was observed in the MI-TLIF group. Postoperative complication rate of the LE-ULBD and the MI-TLIF group were 0 and 5.6%, respectively (*P* = 0.244). One case with transient urinary retention and one case with pleural effusion were observed in the MI-TLIF group. No reoperation was observed within 90 days or during 24 months of follow-up.

**Table 6:** Complications of the two groups.

	**LE-ULBD**	**MI-TLIF**	***P*-value**
*N*	24	36	–
**Intraoperative complications**			
Dural tears	1 (4.2%)	0 (0%)	0.221
Cauda equina injury	0 (0%)	0 (0%)	1.000
Intraoperative complication rate	1 (4.2%)	0 (0%)	0.221
**Postoperative complications**			
Transient urinary retention	0 (0%)	1 (2.8%)	0.414
Pleural effusion	0 (0%)	1 (2.8%)	0.414
Incision fat liquefaction	0 (0%)	0 (0%)	1.000
Incision infection	0 (0%)	0 (0%)	1.000
Implant dislodgement	–	0 (0%)	–
Postoperative complication rate	0 (0%)	2 (5.6%)	0.244

*N indicates number of patients included in the statistical analysis; LE-ULBD, lumbar endoscopic unilateral laminotomy bilateral decompression; MI-TLIF, minimally invasive transforaminal lumbar interbody fusion*.

### Healthcare Cost

The healthcare cost of both groups was listed in Table 7. The surgery cost and anesthesia cost of the LE-ULBD group were less than the MI-TLIF group. Due to the implants used during instrumented fusion, surgical equipment and medical materials cost of LE-ULBD group was significantly less than the MI-TLIF group.

**Table 7:** Healthcare cost of the two groups.

	**LE-ULBD**	**MI-TLIF**	***P*-value**
*N*	24	36	–
Surgery cost	7105.3 ± 853.7 (6,221–8,256)	10602.2 ± 1275.4 (8,999–14,359)	<0.001
Anesthesia cost	3658.8 ± 347.0 (3,489–5,024)	3894.2 ± 437.4 (3,398–5,219)	0.006
Surgical equipment and medical materials cost	3900.0 ± 0.0 (3,900–3,900)	42174.5 ± 8081.1 (33,333–54,245)	<0.001

*N indicates number of patients included in the statistical analysis; LE-ULBD, lumbar endoscopic unilateral laminotomy bilateral decompression; MI-TLIF, minimally invasive transforaminal lumbar interbody fusion; Healthcare Cost was compared in China Yuan (CNY)*.

## Discussion

Even though traditional decompression plus instrumented fusion has been performed to treat patients of LSS with degenerative spondylolisthesis, extensive detaching the paraspinal muscles from the spinous processes and lamina may cause increased intraoperative blood loss, postoperative pain and weakness secondary to muscle denervation. Besides, supraspinous and interspinous ligaments injury and extensive facetectomy may cause iatrogenic spinal instability, requiring additional posterior fixation for stabilization (6). Therefore, various minimally invasive techniques were developed to minimize the surgical trauma (20–22). Patients of LSS with degenerative spondylolisthesis could be treated by both decompression alone and decompression with instrumented fusion (2, 12). LE-ULBD and MI-TLIF are two common minimally invasive procedures to treat LSS with degenerative spondylolisthesis. LE-ULBD and MI-TLIF are typical representative of decompression alone technique and decompression plus instrumented fusion technique, respectively. The present retrospective study revealed that both LE-ULBD and MI-TLIF are effective to treat LSS with degenerative spondylolisthesis. LE-ULBD is a more minimally invasive option for patients of LSS with degenerative spondylolisthesis compared with MI-TLIF, with shorter estimated blood loss, time to ambulation and length of hospitalization. Higher intraoperative complication rate was observed in the LE-ULBD group, while higher postoperative complication rate was observed in the MI-TLIF group. This may be caused by steep learning curve in the LE-ULBD group and greater surgical trauma in the MI-TLIF group.

During LE-ULBD, laminotomy and foraminotomy could be safely performed under excellent endoscopic visualization to guarantee complete decompression, minimize surgical trauma, and prevent iatrogenic spinal instability (13–15). However, sufficient decompression without violating the stability of the facet joints may be technically difficult in cases with narrow interlaminar spaces, posterior marginal osteoproliferation of the vertebrae, ossification of the posterior longitudinal ligaments (13, 14, 23). Excessive facetectomy may be inevitable for sufficient lateral recess decompression and foraminotomy, exacerbating postoperative instability. In the present study, undercutting of the cranial lamina was performed during LE-ULBD to overcome the difficulty during insertion of the working sheath (6). Additionally, the excellent endoscopic visualization achieved during LE-ULBD ensure the undercutting of the cranial lamina, minimized facetectomy, and sufficient decompression of the lateral recess and foramen.

The advantages of LE-ULBD were to perform bilateral decompression *via* a unilateral approach with minimize traumatization to the paraspinal musculoligamentous structures; to ensure the sufficient decompression of the lateral recess and foramen under excellent endoscopic visualization to minimize neurological injury; and to preserve the stability of the spine with minimized foraminotomy (6, 13–15). On the other hand, LE-ULBD has some disadvantages, such as the steep learning curve. Muscles, facet cysts, and ligaments may be difficult to identify under endoscopic visualization, increasing the risk for iatrogenic injury. Besides, traditional open or microscopic surgeries should be performed in necessary if LSS with degenerative spondylolisthesis cannot be sufficiently decompressed during LE-ULBD.

MI-TLIF has been demonstrated to be a safe option for lumbar fusion with minimized iatrogenic traumatization to the paraspinal musculoligamentous structures (24, 25). MI-TLIF was performed to achieve the sufficient decompression of LSS with degenerative spondylolisthesis, immediate improvement of spinal alignment, and prevention of spinal instability (26). MI-TLIF seems to be more safe in cases of LSS with degenerative spondylolisthesis due to higher risk of iatrogenic spinal instability after more extensive foraminotomy (6).

Even though there are growing evidences suggest that decompression alone is of excellent clinical outcome for LSS with degenerative spondylolisthesis, it is still controversial for the necessity of arthrodesis (8, 12, 27–30). Ghogawala et al. (8) found that decompression alone may destabilize the spine in patients of LSS with degenerative spondylolisthesis, resulting in increasing back pain and reoperation. Chan et al. (12) found that MI-TLIF was associated with superior outcomes for disability, back pain, and patient satisfaction and fewer reoperations compared with posterior minimally invasive decompression alone. LE-ULBD could preserve the posterior supporting elements and is recognized to be effective, with the potential to avoid further slip progression. In the present study, there was no significant further slip progression after surgery in both groups. Due to the low complication rate of endoscopic spine surgery, LE-ULBD is getting safer and more reliable. Besides, LE-ULBD is of an economic advantage compared with MI-TLIF.

## Limitations

There are some limitations to the present study. Firstly, it is a retrospective, non-randomized controlled cohort study with a small sample size and short follow-up period. There also may be selection bias, as surgeons determined whether decompression alone or decompress plus instrumented fusion should be performed. Further prospective, randomized, controlled studies, with larger sample sizes and longer follow-up periods should be conducted to determine the optimal surgical management for patients of LSS with degenerative spondylolisthesis. As a result, further studies should be conducted to compare the clinical outcomes of LE-ULBD and MI-TLIF for LSS with degenerative spondylolisthesis. Besides, preexisting adjacent level degeneration was not evaluated and compared in the present study.

## Conclusion

Both LE-ULBD and MI-TLIF are safe and effective to treat one-level LSS with degenerative spondylolisthesis. Although limited by small sample size, short follow-up period, and patients lost to follow-up, this study validates the safety of LE-ULBD as an alternative treatment for one-level LSS with degenerative spondylolisthesis.

## Data Availability Statement

The original contributions presented in the study are included in the article/supplementary material, further inquiries can be directed to the corresponding author/s.

## Ethics Statement

The studies involving human participants were reviewed and approved by Tongji Medical College, Huazhong University of Science and Technology. The patients/participants provided their written informed consent to participate in this study. Written informed consent was obtained from the individuals for the publication of any potentially identifiable images or data included in this article.

## Author Contributions

WH and CY designed the study. WH, BW, WK, and QW collected, assembled, and analyzed the data. Project planning was performed by BW, WK, QX, XW, YZ, and SL. WH wrote the manuscript. All authors read, edited, and approved the manuscript.

## Conflict of Interest

The authors declare that the research was conducted in the absence of any commercial or financial relationships that could be construed as a potential conflict of interest.
