# Corrigendum: Comparison of Clinical Outcomes Following Lumbar Endoscopic Unilateral Laminotomy Bilateral Decompression and Minimally Invasive Transforaminal Lumbar Interbody Fusion for One-Level Lumbar Spinal Stenosis With Degenerative Spondylolisthesis

**DOI:** 10.3389/fsurg.2021.723200

**Published:** 2021-07-30

**Authors:** Wenbin Hua, Bingjin Wang, Wencan Ke, Qian Xiang, Xinghuo Wu, Yukun Zhang, Shuai Li, Shuhua Yang, Qiang Wu, Cao Yang

**Affiliations:** Department of Orthopaedics, Union Hospital, Tongji Medical College, Huazhong University of Science and Technology, Wuhan, China

**Keywords:** lumbar endoscopic unilateral laminotomy bilateral decompression, minimally invasive, transforaminal lumbar interbody fusion, lumbar spinal stenosis, degenerative spondylolisthesis

In the original article, there was some mistakes in Figure 1 and Figure 2 as published (1). Figure 1 is the same as the sketch maps of surgical procedures of lumbar endoscopic unilateral laminotomy bilateral decompression (LE-ULBD) published by us (2). In order to avoid repeated publication of the same figure, we replaced Figure 1. There were also some mistakes in choosing typical intraoperative photos for Figures 2I and 2J. The corrected Figure 1 and Figure 2 appear below.

**Figure 1:**
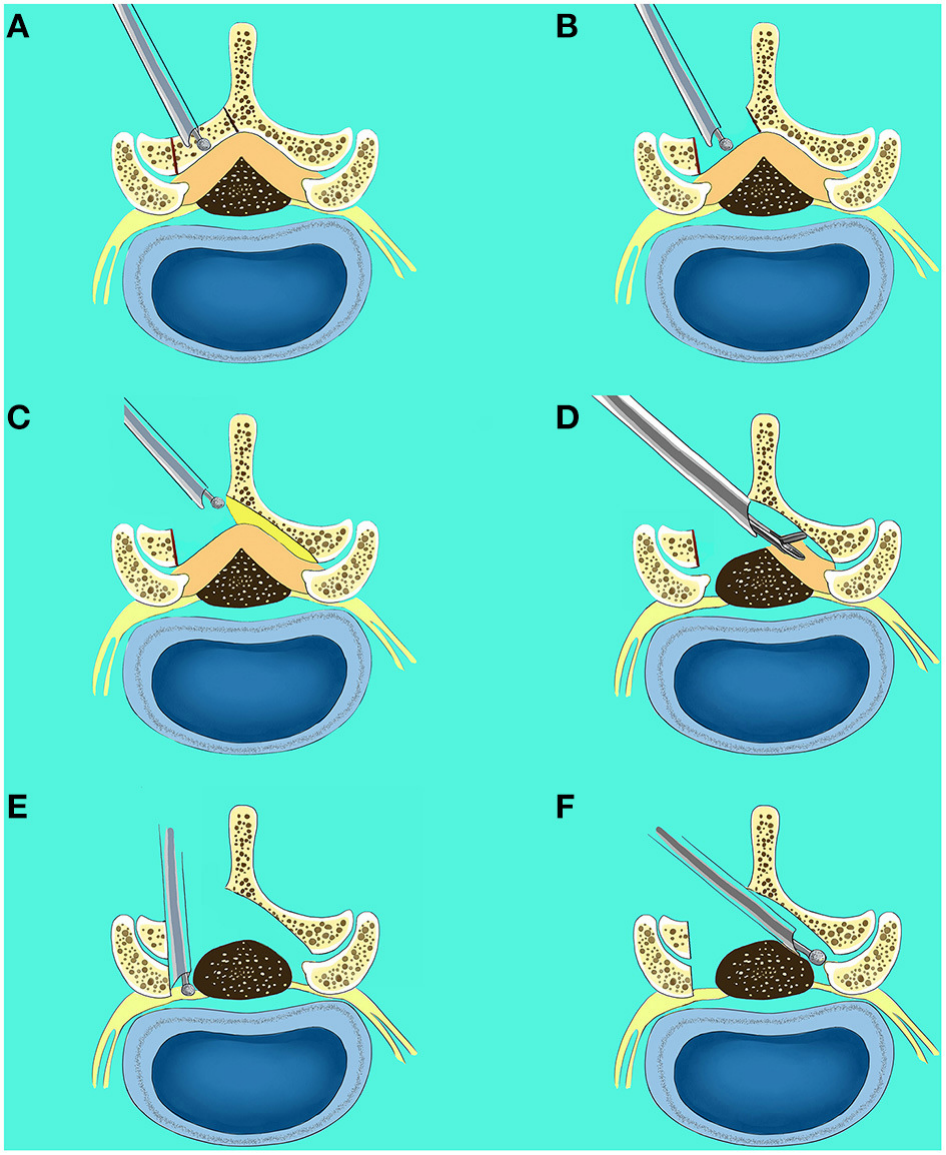
Surgical procedures of lumbar endoscopic unilateral laminotomy bilateral decompression (LE-ULBD). **(A,B)** The inferior edge of the cranial lamina and the base of the spinous process of the ipsilateral side were removed by the endoscopic burr; **(C)** undercutting of the contralateral cranial lamina was performed; **(D)** the ipsilateral and contralateral ligamentum flavum was identified and removed piecemeal with endoscopic punches and forceps; **(E)** the ipsilateral medial facetectomy was performed to decompress the lateral recess and ensure adequate decompression of the traversing nerve root; **(F)** the contralateral medial facetectomy was performed to decompress the lateral recess and ensure adequate decompression of the traversing nerve root.

**Figure 2:**
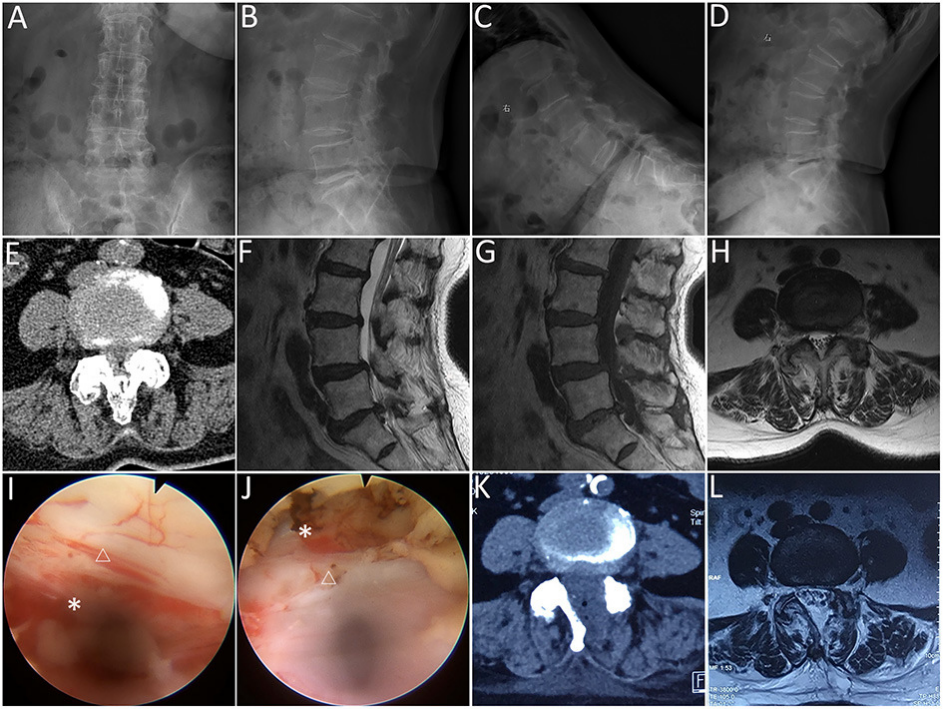
Lumbar endoscopic unilateral laminotomy bilateral decompression (LE-ULBD) performed on a 77-year-old female patient diagnosed with L4-L5 lumbar spinal stenosis with degenerative spondylolisthesis. **(A,B)** preoperative anteroposterior and lateral plain radiographs; **(C,D)** preoperative flexion and extension radiographs; **(E)** preoperative computed tomography (CT) scans; **(F–H)** preoperative magnetic resonance imaging (MRI) scans; **(I,J)** medial facetectomy was performed to decompress the lateral recess and ensure adequate decompression of the traversing nerve root; **(K)** postoperative CT scans; **(L)** postoperative MRI scans. Snowflake, nerve root, triangle, dural sac. * is used to tell the readers where is the nerve root.

The authors apologize for this error and state that this does not change the scientific conclusions of the article in any way. The original article has been updated.

## Publisher's Note

All claims expressed in this article are solely those of the authors and do not necessarily represent those of their affiliated organizations, or those of the publisher, the editors and the reviewers. Any product that may be evaluated in this article, or claim that may be made by its manufacturer, is not guaranteed or endorsed by the publisher.
